# Supplementary material for: Enzyme Properties of a Laccase Obtained from the Transcriptome of the Marine-Derived Fungus Stemphylium lucomagnoense
Source: Int J Mol Sci. 2020 Nov 9;21(21):8402. doi: 10.3390/ijms21218402 (PMC7664933; doi:10.3390/ijms21218402)
Supplement: Supplementary file 1 [file ijms-21-08402-s001.pdf]

```
PsLac2          ---------------------------APQSSAPSLSKRCTNSADDRSCWG-DYDISTNY    32
SlLac2          AVLPVELPNPAPFTPKRDGLEE-RQ-------SPASCINVGHTPKTRHCWAQGFTSSTDM    52
MaLac1          ---------APPSTPAQRDLVELREARQEGGKDLRPREPTCNTPSNRACWSDGFDINTDY    51
PsLac1          ---------------------------AAITQDLEPRQSTCNTASNRRCWTSNFDITTDY    33
                                                         :: . * **  .:  .*:

PsLac2          YEEAPDTGVTREYWFNIVNT--TAAPDGV-ERVVLSVNGSIPGPTIIADWGDTVVVHVTN    89
SlLac2          YTSWPNTGVIRSYNLRLENT--TCNPDGAGSRVCLLINGRFLGPTIVANWGDTIRVTVRN    110
MaLac1          EVSTPDTGVTQSYVFNLTEVDNWMGPDGVVKEKVMLINGNIMGPNIVANWGDTVEVTVIN    111
PsLac1          EQSTPPGG-SVTYNLEITEVRNWKGPDGVVKSFVQLINGQFPGPTIRAKWGDTITVNVKN    92
                  . *  *    * :.: :.     ***. .     :** : **.* *.****: * * *

PsLac2          SMENNGSSIHFHGIRQNYTNQNDGVPSLTQCPIAP-GDSYTYTWRATSYGSSWYHSHFYV    148
SlLac2          NLQSNGTSIHWHGFRMLNKNIQDGVNGITECALAP-GDFKTYQFQATEYGTTWYHSHFSH    169
MaLac1          NLVTNGTSIHWHGIHQKDTNLHDGANGVTECPIPPKGGQRTYRWRARQYGTSWYHSHFSA    171
PsLac1          SMPTNGTSIHWHGLRQMGTNIHDGVNGVTECALAP-GKTRTYRFKATQYGTTWYHSHFSA    151
                .: .**:***:**::   .* :**. .:*:* : * *   ** ::* .**::******

PsLac2          QAWDGIFGGILINGPATANYDEDLGNLFLNDWSHLTADVEVLA-----ALASGPPTLDTG    203
SlLac2          QYGDGVVGTVIVNGPATANYDEDLGVMPITDWYYNTAFQAASIAFQNGQAGLGPPVGDNI    229
MaLac1          QYGNGVVGTIQINGPASLPYDIDLGVFPITDYYYRAADDLVHF-----TQNNAPPFSDNV    226
PsLac1          QYGNGVWGTIVIDGPAENNYDIDLGVYPISDYYYDSADDIVLR-----TQSGGPPASDNV    206
                *  :*: * : ::***   ** ***   :.*: : :*   .           .**  *.

PsLac2          LINGTNVYNE---TGSRYETTFEAGKRYLLRLVNAAADSHFHFSIDNHTLEVIASDFVPI    260
SlLac2          LINGTAKNA--AGGGAWNRVTIQAGKRYRLRLVNTAVDTNMLVNLDGHPFQVIATDFVPI    287
MaLac1          LINGTAVNPN-TGEGQYANVTLTPGKRHRLRILNTSTENHFQVSLVNHTMTVIAADMVPV    285
PsLac1          LFNGTNINPLNPSQGKYSVVTLTPGKRHRLRLINPSVEHNYQVSLVGHDMTVIATDLVPV    266
                *:***         *    .*:  ***: **::* :.: :  ..: .* : ***:*:**:

PsLac2          EPYTTDVVSIAMGQRYDVIVTANATADNYWMRALI--QTACSDNANPDNVRGIIRYDSTS    318
SlLac2          NPYNTTHLQIGIGQRYDVIINANQTAGNYWFRATA--DGLCQSRNARE-GRAIFTYQGQP    344
MaLac1          NAMTVDSLFLAVGQRYDVVIDASRAPDNYWFNVTFGGQAACGGSLNPH-PAAIFHYAGAP    344
PsLac1          NSMTLNNIYLGTGQRYDVLIDASKTVGNYWFNVTLSPTGLCGTSINPR-PAAIFRYAGAP    325
                :  .   : :. ******:: *. : .***:..       *          .*: * .

PsLac2          TADPTSTAWDDATTDGTCYDEDISQLVPYVPITASST--ADYEDDFSV---KIVVGDNGV    373
SlLac2          VADPTSTAPT--VPFTACLDPVT--T-PKIVKNVPSSTFAAQAKNLPVAFGPVAANGNTV    399
MaLac1          GGLPTDEGTP--PVDHQCLDTLD--VRPVVPRSVPVNSFVKRPDN-TLPVALDLTGTPLF    399
PsLac1          STNPTDPGTV--PADAACQDRTD--FTPVVTRTADSTTLVPQANDPAHNLDISLNFTTPV    381
                   **. .         * *       * :  ..  .  .   .:              .

PsLac2          LWEMGPSSFVNQWDYPSLLQVEEGNDTWADSQQVYAFPD--ADTWVYWVIQTT----NNQ    427
SlLac2          LWTINGTSMIIDPGKPTIKYVAENNNSFPQSYNVVKVPATSASTWTYWVIQQA-VGAPPI    458
MaLac1          VWKVNGSDINVDWGKPIIDYILTGNTSYPVSDNIVQVDA--VDQWTYWLIENDPEGPFSL    457
PsLac1          SWFVNGSAIDVQWDKPVLDYVLTGNTSYPRRENLVFVDD--KNVWTYWVVQNL----SPL    435
                 * :. : :  : . * :  :  .* ::    ::  .     . *.**:::

PsLac2          PHPMHLHGHDFWILGQGLGTYD-----------ADTANLTYTNPPRRDVVQLPGAGYVAI    476
SlLac2          AHPIHLHGHDSYILGAGDGQ---------FNAATHFSQLRFTNPPRRDVSQLKGNGWLVI    509
MaLac1          PHPMHLHGHDFLVLGRSPDVPAASQQRFVFDPAVDLARLNGDNPPRRDTTMLPAGGWLLL    517
PsLac1          PHPMHLHGHDFLVLGHAASSTFTA---------SMSSSLNFNNPTRRDVTMLPASGYVIL    486
                 **:******  :**  . .                 : *   ** ***.  *  . *:: :

PsLac2          AFYTDNPGVWLMHCHIAWHTSEGLAVQVLERESEIGALID---ADVMNSTCAAWDDYTAE    533
SlLac2          AYPTDNPGAWLMHCHIAFHVGMGLSVQFLERKQQIV---LPATGSEWYNNCNNWAAYKAG    566
MaLac1          AFRTDNPGAWLFHCHIAWHVSGGLSVDFLERPADLRQRISQEDEDDFNRVCDEWRAYWPT    577
PsLac1          AFKADNPGNWLMHCHIAWHVSGGLSVDFMERRAEQAALISAADLAAYKQQCADWRAYAPK    546
                *: :**** **:*****:*.. **:*:.:**  :                *  *  *

PsLac2          --DDIIQDDSGI-----------------  543
SlLac2          NTDVWPQDDSGLKKR-WPPLIEDGSVL--  592
MaLac1          --NPYPKIDSGLKRRRWVE---ESEWLVR  601
PsLac1          --AP-PKIDSGI-----------------  555
                      : ***:
```

Figure S1: Alignments of the closest characterized laccases from *Melanocarpus albomyces* (*Ma*Lac1, Q70KY3) and *Pestalotiopsis* sp. KF079 (*Ps*Lac1: KY554800 and *Ps*Lac 2: KY554801), using CLUSTAL W sequence alignment algorithm. Perfect matches are represented by an asterisk, high-amino acid similarities by double dots, and low-amino acid similarities by single dots. Gaps (-) were introduced for maximum alignment. The amino acids involved in the coordination sites for the type 1, 2 and 3 coppers are in red and bold.

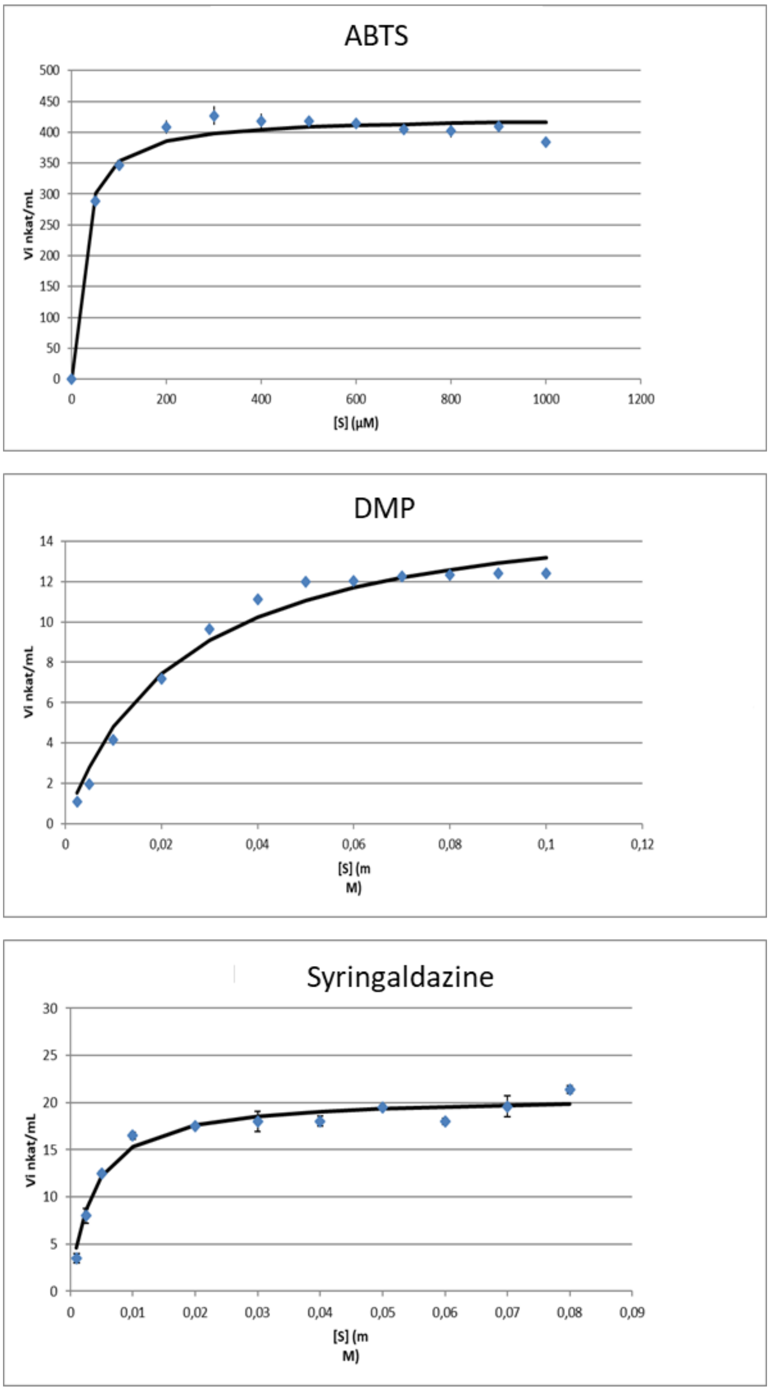

Figure S2: Kinetics of *Sl*Lac2 on the three substrates.
